# Prediction of anxiety in children aged 7–12 during preoperative anaesthesia evaluation – a prospective observational study

**DOI:** 10.1186/s13741-026-00669-2

**Published:** 2026-03-12

**Authors:** Armin Sablewski, Katarina Krebs, Anna Karstensen, Maximilian Grosser, Charlotte Neitzel, Axel Fudickar, Barbara Schmidt, Tobias Becher

**Affiliations:** 1 Department of Anaesthesiology and Intensive Care Medicine, University Medical Center Schleswig-Holstein, Campus Kiel, Kiel, Germany; 2 Institute for Psychosocial Medicine, Psychotherapy and Psychooncology, University Hospital, Jena, Germany

**Keywords:** Paediatric anaesthesia, Preoperative anxiety, Anxiety prediction, Preoperative assessment, mYPAS

## Abstract

**Background:**

Preoperative anxiety is a common challenge in paediatric anaesthesia. High anxiety levels are associated with adverse perioperative outcomes. Although risk factors for preoperative anxiety in children are well known, validated assessment tools are rarely used in everyday clinical practice. Instead, anaesthesiologists rely on clinical judgment during preoperative assessments. However, the accuracy of such anxiety predictions in clinical practice remains unclear.

**Methods:**

This prospective observational study included children aged 7–12 years undergoing elective surgery or diagnostic procedures under general anaesthesia. During the preoperative consultation, anaesthesiologists, parents, and children were asked to predict the child’s anxiety on a Visual Analogue Scale (VAS), while actual anxiety during anaesthesia induction was assessed using the Modified Yale Preoperative Anxiety Scale – Short Form (mYPAS-SF). Spearman’s correlations (r_s_) between predicted and observed anxiety were calculated, and the predictive performance for clinically significant anxiety (defined as mYPAS-SF > 30) was evaluated using the area under the receiver operating characteristic curve (AUC). Potential predictors of preoperative anxiety, including demographic and psychosocial factors, were also evaluated.

**Results:**

91 datasets sets were analysed. Anxiety prediction by parents correlated moderate to children’s anxiety during anaesthesia induction (AUC 0.661; r_s_=0.327, 95% CI 0.12–0.51). Anxiety predictions by anaesthesiologists (AUC 0.604; r_s_=0.173, 95% CI -0.04–0.37) and children (AUC 0.612; r_s_=0.147, 95% CI -0.08–0.36) correlated weakly with children’s anxiety. Children reporting being worried before anaesthesia showed significantly higher anxiety during induction (mYPAS-SF 38.5 [22.9–57.3] vs. 22.9 [22.9–38.0]; *P* = 0.006). Children’s anxiety at consultation and on the ward correlated with increased anxiety during induction (r_s_=0.271, 95% CI 0.06–0.46 and r_s_=0.297, 95% CI 0.09–0.48, respectively). Intravenous induction was associated with lower anxiety compared with inhalational induction (mYPAS-SF 22.9 22.9 [22.9–38.0] vs. 38.5 [22.9–57.3]; *P* < 0.001). No significant associations were found for age, gender, prior medical experiences, or parental anxiety.

**Conclusions:**

Clinical anxiety assessments in preoperative consultations are often inaccurate, with parents performing slightly better than anaesthesiologists and children. Structured screening approaches are needed to enhance the early identification of high-risk children and improve perioperative anxiety management.

**Trial registration:**

German Clinical Trials Registry, registration number DRKS00033395. Date of registration: 06/05/2024.

**Supplementary Information:**

The online version contains supplementary material available at 10.1186/s13741-026-00669-2.

## Introduction

Preoperative anxiety is a common phenomenon in paediatric patients, with reported incidence rates ranging from 40% to 70% (Liu et al. 2022). The intensity of anxiety varies widely and is associated with maladaptive postoperative outcomes (Kain et al. 2004; Banchs and Lerman 2014). Addressing preoperative anxiety is therefore a significant challenge in paediatric perioperative medicine and is recognized as a key component of the “10-N Quality Criteria” for paediatric anaesthesia (Weiss et al. 2015). Research priorities of consumers revealed a strong interest in anxiety reduction (Sommerfield et al. 2023).

Perioperative anxiety can be assessed using the Modified Yale Preoperative Anxiety Scale (mYPAS) and its short form (mYPAS-SF), both widely used observational tools (Jenkins et al. 2014). In clinical practice, the visual analog scale (VAS) is often preferred for its simplicity and ease of use (Bringuier et al. 2009; Berghmans et al. 2017). Both pharmacological and non-pharmacological interventions can be used to manage perioperative anxiety (Calipel et al. 2005; Manso et al. 2019; Bromfalk et al. 2021).

Several factors have been linked to increased preoperative anxiety, including psychological predispositions, particularly internalizing behaviours, younger age, temperamental disposition, high parental anxiety, language barriers, and negative prior hospital experiences (Kain et al. 2006; Berghmans et al. 2015; Sola et al. 2017; Mamtora et al. 2018); Chow et al. 2019; Getahun et al. 2020). Although standardized psychological tests like the Strengths and Difficulties Questionnaire (SDQ), Emotionality, Activity, and Sociability Temperament Survey (EASI), Child Behavior Checklist (CBCL), or State-Trait Anxiety Inventory for Children (STAIC) provide valuable insights, they are not routinely used in preoperative evaluations (Papay and Spielberger 1986; Goodman 1997; Fortier et al. 2010; Berghmans et al. 2015).

Despite ongoing research efforts, preoperative anxiety remains a persistent challenge in clinical practice. First, preoperative anxiety is often not systematically assessed by clinicians, leading to underrecognition and potentially inadequate management (Sablewski et al. 2025). Second, the preoperative evaluation typically occurs one to several days before surgery, creating a time lag between assessment and anaesthesia. At this stage, the child is not yet exposed to procedural stress and usually remains calm in the presence of their parents. However, anxiety tends to increase during the inpatient stay, often peaking at anaesthesia induction (Kain et al. 2007). This gap in assessment means that clinicians lack insight into how the child may react during induction of anaesthesia at a higher stress level and in unknown surroundings. Third, preoperative evaluations primarily focus on somatic risks related to anaesthesia, partly due to legal requirements (Fletke et al. 2022). As a result, anaesthesiologists must rely on their clinical impression to identify children at high risk for severe anxiety and decide on appropriate interventions, such as premedication.

The aim of this study was to investigate whether anaesthesiologists, parents and the children themselves were able to predict the preoperative anxiety of paediatric patients during induction of anaesthesia when asked during the preoperative evaluation. To further improve anxiety prediction, we also sought to identify independent early predictors of increased anxiety during induction of anaesthesia.

## Methods

### Study design

This prospective monocentric observational trial was registered at the German Clinical Trials Registry (date of first trial registration 06/05/2024, registration number.

DRKS00033395), https://drks.de/register/en/trial/DRKS00033395/preview and was approved by the local Institutional Review Board (Ethics Committee of Christian- Albrechts-University of Kiel, Germany, ethics approval number D418/21). This observational cross-sectional cohort study was set up according to the Equator Network STROBE guidelines (von Elm et al. 2007). This study was performed in accordance with the relevant guidelines and regulations as well as in accordance with the Declaration of Helsinki.

### Setting and participants

The study was conducted at the University Medical Center Schleswig-Holstein (UKSH), Kiel Campus, Germany, between March 2024 and January 2025. Screening was performed daily on weekdays by the study team at the preoperative anaesthesia clinic. The number of screened and included patients, along with reasons for non-inclusion, were documented anonymously in a pre-screening log.

Children aged 7 to 12 years scheduled for elective procedures (surgery or diagnostic examination) requiring general anaesthesia were eligible. Exclusion criteria were language barriers (for parents, legal guardians, or children) as well as previous participation in this study. Written informed consent was obtained from the parents or legal guardians for all participants. 

### Study protocol

During preoperative evaluation, demographic data and baseline measures were collected along with information on the child’s and parental native language and migration status. Parents rated their current anxiety using the VAS before receiving information about the planned anaesthesia. Preoperative information about the planned anaesthesia procedure was provided in accordance with institutional standards, using structured information sheets and allowing time to address individual questions. After informed consent for the planned procedure was obtained anaesthesiologists, parents, and children predicted the child’s anxiety level during anaesthesia induction in the operating theatre using the VAS. Simultaneously, the study team assessed the child’s anxiety using the mYPAS-SF.

A coded note was documented to ensure that the anaesthesiologist performing the preoperative evaluation differed from the provider responsible for anaesthesia on the day of surgery. Children were asked an open-ended question to name a word or place where they feel comfortable or safe. Their responses were recorded and subsequently categorized for qualitative analysis.

On the day of surgery, the child’s anxiety was assessed on the ward using the mYPAS-SF prior to midazolam administration. In accordance with standard hospital protocol, patients received 0.5 mg/kg of oral midazolam preoperatively. In the ambulatory surgery unit and for MRI examinations, premedication with midazolam was not prescribed routinely to allow for rapid recovery and discharge. However, administration was possible at the discretion of the attending anaesthesiologist in cases of notable preoperative distress. A local anaesthetic cream with Lidocaine and Prilocaine (EMLA, Aspen Pharmacare, Durban, South Africa) was applied to an appropriate skin area 30–60 min before transfer to the operating theatre or examination area. Parents did not accompany their children during anaesthesia induction, remaining instead in the holding area - except for procedures in the ambulatory surgery unit or for MRI examinations. All anaesthesiologists were familiar with the hospital’s protocols and preoperatively conveyed this information to the participants. Children complied with the perioperative fasting guidelines established by the European Society of Anaesthesiology and Intensive Care (Frykholm et al. 2022).

**Fig. 1:**
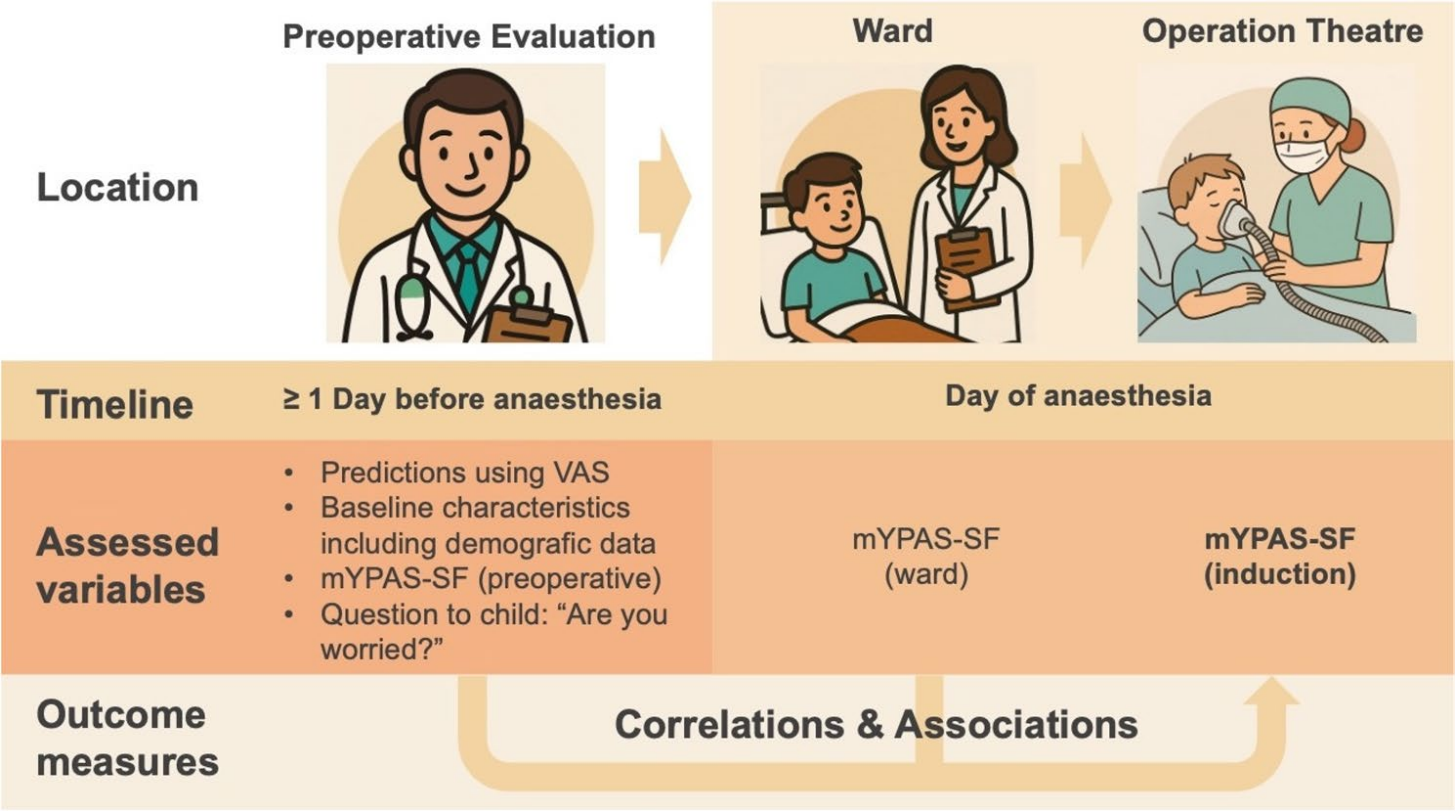
Schematic overview of data collection points and outcome assessment across the three clinical phases. Abbreviations: VAS Visual Analog Scalle; mYPAS-SF Short Form of the Modified Yale Preoperative Anxiety Scale

Anaesthesia preparation, including the placement of electrocardiography electrodes, a pulse oximeter, and a blood pressure cuff, was performed before anaesthesia induction in the operating theatre. Anaesthesia was induced intravenously (IV) whenever possible. For IV induction, a peripheral IV catheter was inserted through an anesthetized area of the skin, followed by the administration of propofol (2–5 mg/kg IV) and sufentanil (0.25–0.5 mcg/kg IV). If IV line placement was not preferred or unsuccessful, inhalation induction was performed instead. Sevoflurane in a mixture of oxygen and air was administered via mask, with IV line placement occurring after induction. In both settings (IV or inhalation induction), children’s anxiety was assessed using the mYPAS-SF during preoxygenation with mask. Anaesthesia induction was performed by a board-certified anaesthesiologist or under their supervision, with all staff instructed to interact with children in a supportive and age-appropriate manner. Figure 1 provides an illustrated overview of the study design.

### Data sources/measurement

The study team underwent standardized training to ensure consistent scoring and data integrity and gathered all measures. Training consisted of several weeks of supervised clinical practice until consistent parameter collection, particularly of the mYPAS, was achieved. Once their scoring was deemed consistent with the experienced raters, they were allowed to perform independent assessments. Whenever possible, the same two raters assessed the mYPAS-SF. Interrater reliability was assessed using the intraclass correlation coefficient (ICC) for all cases in which both raters provided ratings.

The Short Form of the Modified Yale Preoperative Anxiety Scale (mYPAS-SF) was used to assess children’s anxiety during preoperative evaluation, on the ward, and at anaesthesia induction. The mYPAS-SF is an observational measure of preoperative anxiety consisting of 18 items in four domains (activity, emotional expressivity, state of arousal, and vocalization). The mYPAS-SF total score ranges from 22.92 to 100, with higher scores indicating greater anxiety.

A Visual Analog Scale (VAS) was used for the prediction of children’s anxiety (VAS-anxiety). The VAS is a 100 mm horizontal line that pictorially represents two behavioural extremes at either end of a continuum. Anaesthesiologists, parents and children placed a mark on the line to indicate how anxious they thought the child would be when s/he got the mask for preoxygenation (defined here as anaesthesia induction). Scores were rounded up to a range from 0 to 10, with higher scores indicating higher levels of predicted anxiety.

The German version of the MacArthur Scale was used to measure subjective social status (SSS). Parents were asked to place themselves on a 10-rung ladder (ranging from 1 to 10), with higher rungs indicating higher income, education, and job status relative to others in Germany (Adler et al. 2000; Hoebel et al. 2015).

Parents and children were asked to rate their perception of the planned medical procedure using a 3-point Likert scale (small, medium, large). Both an objective assessment of the expected surgical / interventional trauma (“How would you rate the extent of the procedure?”) and a subjective assessment of the intervention’s personal significance (“How significant is the procedure for you personally?”) were requested. Children were asked whether they were worried about the upcoming procedure, and their dichotomous responses (yes/no) were recorded. 

### Statistical methods

The primary analysis focused on the Spearman correlation (r_s_) between anxiety predictions, assessed using the Visual Analog Scale (VAS) during the preoperative consultation, and children’s anxiety during anaesthesia induction, measured with the Modified Yale Preoperative Anxiety Scale - Short Form (mYPAS-SF). Based on prior research suggesting a correlation of 0.3 (MacLaren et al. 2009), we calculated the required sample size using G*Power (version 3.1.9.6). Assuming a two-sided significance level of 0.05 and a power of 0.80, we determined that 89 prediction pairs were needed. To compensate for potential dropouts, we increased the target recruitment by 5%, setting a final goal of 94 patients. To account for multiple testing in the correlation analyses (predictions by anaesthesiologists, parents, and children), the Bonferroni correction was applied, setting the significance threshold at *P* < 0.017 for the three pairwise comparisons. Additionally, the predictive performance for clinically relevant actual anxiety during anaesthesia induction (defined as mYPAS-SF > 30) was assessed by calculating the area under the receiver operating characteristics curve (AUC).

In the secondary analysis, we examined the association between various continuous and categorical predictor variables and children’s anxiety during anaesthesia induction. Continuous predictors included the child’s age, parents’ age, preoperative mYPAS-SF, ward mYPAS-SF, parental anxiety (VAS), and family Subjective Social Status SSS, which was defined as the mean of both parents’ SSS scores when both were present. Categorical predictors comprised variables assessed during the preoperative evaluation setting (child’s gender, prior experience with anaesthesia, treatment setting, native language of the mother, father, and child, the child’s migration status, and the child’s self-reported anxiety [“Are you worried?“]) as well as variables assessed during the anaesthesia induction setting (type of anti-anxiety intervention, type of anaesthesia induction, and the presence of a parent during induction).

Children’s feel-good places and the perceptions of both parents and children regarding the planned medical procedure were analysed exploratively.

In the descriptive analysis, baseline characteristics were presented as mean (SD) for normally distributed continuous variables, and as median (interquartile range [25th–75th percentile]) for non-normally distributed continuous variables. Categorical variables were reported as absolute and relative frequencies. The Kolmogorov-Smirnov test was used to evaluate the normality of data distribution. To assess potential associations between predictor and outcome variables, univariate analyses were performed. Categorical variables were analysed using the Fisher’s exact test or chi-square test, as appropriate. Continuous variables were compared using the Mann–Whitney U test for non-normally distributed data. All tests were two-sided, with P-values ≤ 0.05 considered statistically significant. Analysis was based on complete cases; missing data were logged separately.

We calculated the intraclass correlation coefficient (ICC) among patients rated by the same two raters to assess interrater reliability. ICC was computed using a two-way random-effects model with absolute agreement for single measures (model = “twoway”, type = “agreement”, unit = “single”) in the irr package in R.

Analysis and illustrations were performed using GraphPad Software, LaJolla California, USA) and Microsoft Excel (Office 2019, Microsoft, Redmond, USA) and R version 4.5.1 (R Foundation for Statistical Computing, Vienna, Austria). Images were generated by AI (DALL·E, OpenAI, ChatGPT; June 2025, USA) without using any original or copyrighted content.

## Results

### Baseline characteristics

**Fig. 2:**
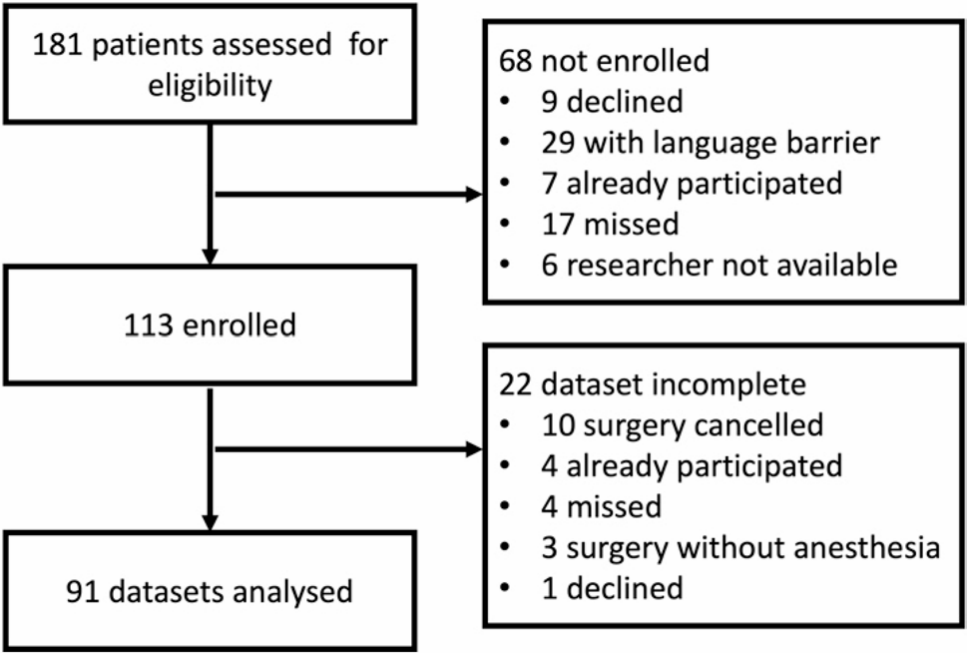
Participant workflow diagram for full-analysis set

A total of 181 patients aged 7 to 12 years met the inclusion criteria, of whom 91 were included in the study (full analysis set, see Fig. 2). Unless stated otherwise, all data and results refer to this primary analysis population. The baseline characteristics of the included patients are presented in Table 1. The trial concluded as planned in January 2025, having successfully recruited the targeted number of participants.

**Table 1:** Characteristics of the children and accompanying parent(s)

Characteristic	Total (*N* = 91)
Age, year	9 [8–10]
Male	49 (54%)
Female	42 (46%)
Weight, kg	32 [25–42]
Height, cm	136 [128–149]
ASA	
I	64 (70%)
II	16 (18%)
III	11 (12%)
Reason for anaesthesia	
Surgery	84 (92%)
Diagnostics	7 (8%)
Treatment setting	
Outpatient (daycase)	56 (62%)
Hospitalized	35 (38%)
First hospital experience, n (%)	74 (81%)
First anaesthetic experience, n (%)	69 (76%)
Type of Surgery/Intervention	
ENT	14 (15%)
Oral and Maxillofacial Surgery (OMFS)	9 (10%)
Urology	4 (4%)
General Surgery	20 (22%)
Ophthalmology	22 (24%)
Paediatrics (MRI Diagnostics)	8 (9%)
Orthopaedic surgery	14 (15%)

Values are presented as median [25th–75th percentile] or number (proportion)

Abbreviations: *MRI *magnetic resonance imaging

### Preoperative evaluation setting

Family background characteristics of parents and children are shown in Table 2.

**Table 2:** Family Background Characteristics of Parents and Children

Category	Variable	Values
Child accompanied by	Mother	69 (67%)
	Father	15 (17%)
	both	14 (16%)
Parents age	Fathers	44.7 ± 12.3
	Mothers	39.6 ± 7.1
Migration background child	Yes	26 (29%)
	No	65 (71%)
Mother’s native language	German	66 (73%)
	Non-German	25 (27%)
Father’s native language	German	65 (73%)
	Non-German	24 (27%)
Children’s native language	German	70 (77%)
	Non-German	21 (23%)
Subjective social status [1–10]	Mother	5.8 ± 1.7
	Father	5.6 ± 1.8

Values are presented as mean ± SD or number (proportion)

The professional experience of the anaesthesiologists for the preoperative evaluation ranged from 0 to 30 years, with a median of 2 years [0.5–5]. Among them, 18 (20%) were board-certified specialists. In terms of premedication, midazolam was prescribed in 64 instances (70%), clonidine in 1 (1%), and no medication was prescribed in 26 instances (29%). For midazolam, the prescribed median dose was 8.25 mg [0–12] or 0.35 mg/kg [0.22 – 0.48].

Parental anxiety before the evaluation was 4 [1–6], measured using the VAS. The median mYPAS-SF scores of children’s anxiety was 22.9 [22.9–22.9] during preoperative evaluation.

Children and parents were asked to evaluate the objective and subjective magnitude of their medical procedure based on the surgical trauma. Among the 62 responding children, 31 classified the procedure as objectively small, 23 as medium, and 8 as large. When assessing the personal significance of the procedure, 68 children participated, with 19 considering it small, 28 medium, and 21 large. The difference between objective and subjective assessments was significant (*P*=0.018) for children. A total of 86 parents rated the objective magnitude of the surgery, with 53 categorizing it as small, 27 as medium, and 6 as large. In contrast, when evaluating their personal perception, 91 parents responded, with 23 perceiving the procedure as small, 21 as medium, and 47 as large. The difference between objective and subjective assessments was highly significant (*P*<0.001) for parents.

While there was no statistically difference between the objective assessments of parents and children (*P*=0.301), their subjective evaluations showed a difference between parents and children with parents perceiving the procedure as significantly larger than children (*P*=0.018).

As part of the assessment, children were invited to mention words or places associated with comfort or safety. The full list of responses is available in an additional table (see Additional File 1). 

### Anaesthesia induction setting

The median mYPAS-SF score was 22.9 [22.9–32.8] on the ward before anti-anxiety interventions (pharmacological and/or non-pharmacological interventions). Interrater reliability between the two raters was 0.96 (n=10, 95 % CI 0.86–0.99; *P*<0.001).

The median professional experience of anaesthesiologists was 6 years [4–10.3]. Parental presence during induction was reported in 39 instances (42.9%). Non-pharmacological interventions were applied in 57 instances (62.6%). A detailed list of applied interventions is available in Additional File 2. Regarding pharmacological interventions, Midazolam was given in 43 instances with a median dose of 11.0 mg [7.5–15] or 0.32 mg/kg [0.27 – 0.45]. Regarding sedation levels prior to induction of anaesthesia, 62 patients (68%) showed no sedative effect, 23 (25%) were calm, and 6 (7%) were asleep. 24 patients (26%) underwent inhalational induction, while 67 patients (74%) received intravenous induction. 

### Accuracy of anxiety prediction

The median mYPAS-SF score was 29.2 [22.9–50.0] during the induction of anaesthesia. Interrater reliability between the two raters was 0.97 (n=17, 95 % CI 0.93–0.99; *P*<0.001).

The analyses included 91 prediction sets for anaesthesiologists, 80 for children, and 89 for parents. Pairing effectiveness, evaluated using Spearman’s correlation coefficient (rs), yielded values of rs=0.173 for anaesthesiologists (95% CI -0.041–0.37, *P*=0.102), rs=0.147 for children (95% CI -0.082–0.36, *P*=0.194), and rs=0.327 for parents (95% CI 0.12–0.51, *P*<0.002), with significant correlation observed only in the parent group. This correlation also remained significant after Bonferroni correction for multiple testing (*P*<0.017).

**Fig. 3:**
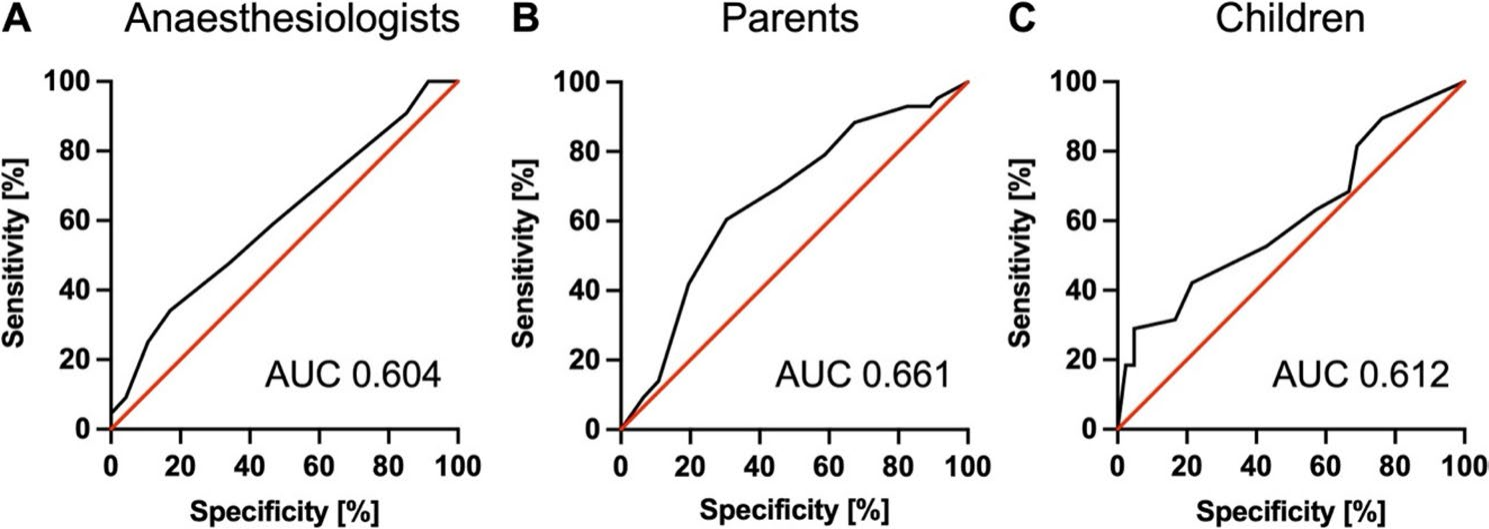
ROC curves for prediction of preoperative anxiety by anaesthesiologists (**A**), parents (**B**), and children (**C**). Abbreviations: AUC, area under the receiver operating characteristics curve

The AUC for predicting clinically relevant anxiety during anaesthesia induction was 0.604 (95% CI: 0.488–0.720, SE: 0.0593, *P*=0.089) for anaesthesiologists, 0.612 (95% CI: 0.487–0.736, SE: 0.0636, *P*=0.086) for children, and 0.661 (95% CI: 0.547–0.775, SE: 0.058, *P*=0.001) for parents (see Figure 3).

In all cases, the anaesthesiologists assessing the child during the preoperative evaluation differed from the provider administering anaesthesia on the day of surgery.

### Predictor variables for children’s anxiety

Univariate results are presented in Table 3 and Table 4. Spearman’s correlation demonstrated that higher levels of anxiety during preoperative evaluation and on the ward were associated with higher levels of anxiety during anaesthesia induction (see Table 3).

Among the preoperative evaluation parameters, the child's response to the question “Are you worried?” was associated with increased anxiety levels (median mYPAS 38.5 [22.9–57.3] for “yes” vs. 22.9 [22.9–38.0] for “no”; *P* = 0.006).

During anaesthesia induction, IV induction was linked to lower anxiety, while no other parameters showed relevant differences (see Table 4).

**Table 3:** Univariate associations between continuous predictor variables and children’s anxiety during anaesthesia induction

Predictor variables	Spearman *r*_s_	95% CI	*P*-Value
Child age	-0.127	-0.330 to 0.088	0.232
Parents age	-0.119	-0.323 to 0.096	0.262
mYPAS-SF (preoperative)	0.271	0.063 to 0.457	0.009
mYPAS-SF (ward)	0.297	0.087 to 0.482	0.005
Parental anxiety	0.101	-0.114 to 0.306	0.343
Family SSS	0.044	-0.172 to 0.256	0.683

Abbreviations: *mYPAS-SF* Short Form of the Modified Yale Preoperative Anxiety Scale, *SSS *Subjective Social Status assed with the German Version of the MacArther Scale

**Table 4:** Univariate associations between categorical predictor variables and children’s anxiety during anaesthesia induction

Category	mYPAS-SF	*P*-Value
Preoperative Evaluation Setting		
Gender		
Male (*n* = 49)	33.3 [22.9–46.9]	0.796
Female (*n* = 42)	27.1 [22.9–52.1]	
Prior Experience		
With Hospitals		
Yes (*n* = 74)	29.2 [22.9–48.4]	0.484
No (*n* = 17)	33.3 [22.9–56.3]	
With Anaesthesia		
Yes (*n* = 69)	27.1 [22.9–46.9]	0.103
No (*n* = 22)	37.5 [22.9–57.3]	
Treatment Setting		
Hospitalized (*n* = 35)	29.2 [22.9–50.0]	0.954
Daycase (*n* = 56)	29.2 [22.9–47.9]	
Native Language		
Mother		
German (*n* = 66)	27.1 [22.9–50.5]	0.312
Non-German (*n* = 25)	38.5 [22.9–45.8]	
Father		
German (*n* = 65)	29.2 [22.9–51.0]	0.528
Non-German (*n* = 24)	38.5 [22.9–45.8]	
Child		
German (*n* = 70)	28.1 [22.9–50.0]	0.401
Non-German (*n* = 21)	35.4 [22.9–51.0]	
Migration Status (child)		
No (*n* = 26)	37.0 [22.9–45.8]	0.444
Yes (*n* = 65)	27.1 [22.9–51.0]	
Are you worried? (child)		
Yes (*n* = 49)	38.5 [22.9–57.3]	0.006
No (*n* = 36)	22.9 [22.9–38.0]	
Anaesthesia Induction Setting		
Anti-anxiety intervention		
pharmacological		
Yes (*n* = 43)	29.2 [22.9–50.0]	0.879
No (*n* = 46)	31.3 [22.9–48.4]	
Non-pharmacological		
Yes (*n* = 57)	33.3 [22.9–57.3]	0.137
No (*n* = 34)	28.1 [22.9–42.3]	
Induction of anaesthesia		
IV (*n* = 67)	22.9 [22.9–41.7]	< 0.001
Inhalative (*n* = 24)	52.1 [37.0-88.5]	
Parental presence		
Yes (*n* = 39)	39.6 [22.9–60.4]	0.072
No (*n* = 52)	27.1 [22.9–44.1]	

Values are presented as median [25th–75th percentile]

Abbreviations: *mYPAS-SF* Short Form of the Modified Yale Preoperative Anxiety Scale, *IV *intravenous

## Discussion

This study aimed to evaluate the accuracy of anxiety predictions made by anaesthesiologists, parents, and children, and to identify key predictors of heightened anxiety during anaesthesia induction. Our findings suggest that anaesthesiologists and the children themselves are unable to make a reliable prediction of preoperative anxiety, while parents predicted children’s anxiety during anaesthesia induction with moderate accuracy. The correlation between preoperative and induction anxiety, together with the child’s response to the simple yes/no question “Are you worried?”, represent potential early predictors of preoperative anxiety.

Our findings partially align with those of MacLaren et al. (2009) but differ in methodology. Unlike our study, their predictive assessments were conducted in the holding area, closer to the time of surgery. The previous study distinguished between attending and resident anaesthesiologists reporting higher predictive accuracy among attendings. Our study primarily involved resident anaesthesiologists and their ability to predict anxiety remained poor. This reflects daily clinical practice in Germany, where preoperative consultations tend to be performed by younger physicians. It remains unclear how the "gut feeling" of experienced attending anaesthesiologists could be systematically integrated into anxiety assessments — due to its potential importance for targeted anxiety prevention. Interestingly, parents in our study showed higher predictive accuracy than those in MacLaren et al.’s study.

In another study, fathers seemed to be more accurate in prediction of children anxiety than mothers (Thompson et al. 2009). 

To our knowledge, few studies have assessed children's self-predictions of preoperative anxiety. In paediatric populations aged 7 to 12 years, this developmental stage is characterized by an increased ability to engage in preoperative preparation. It was therefore of particular interest to examine whether children could act as their own experts in predicting their anxiety levels. While their estimated anxiety levels did not correlate with observed induction anxiety, a simple dichotomous question (“Are you worried?”) was significantly associated with anxiety levels. This suggests that binary formats may be more accessible and reliable for young patients.

Our study suggests that pre-existing increased anxiety levels of children are predictors of anxiety at induction, consistent with previous research highlighting trait anxiety as a key determinant of procedural distress (Eijlers et al. 2021; Cheng et al. 2023). However the anxiety was assessed with the mYPAS-SF and its application is not widely used in clinical practice, although, the scoring procedure itself is not overly complex. It would therefore be desirable for the early recognition of preoperative anxiety to become a systematic part of preoperative assessment — ideally through the inclusion of a brief anxiety screening or question — as this is currently not routine and is documented in fewer than 5% of all consultations (Sablewski et al. 2025).

Parental anxiety has also been linked to increased child anxiety (Kain et al. 2004; Davidson et al. 2006; Kain et al. 2006; Fortier et al. 2010; Cui et al. 2016). Previous studies have typically assessed parental anxiety using the State-Trait Anxiety Inventory (STAI), whereas our study adopted a pragmatic approach with a visual analog scale, finding no correlation between parental and child anxiety. The VAS has been widely used to assess subjective states such as preoperative anxiety (Kindler et al. 2000; Davey et al. 2007; Berghmans et al. 2017). In our study, parental anxiety was measured at least one day before surgery, based on the assumption that early anxiety might influence the child’s emotional state. However, no association was found between early parental anxiety levels and the child’s anxiety during anaesthesia induction. This may be explained by a temporal gap between assessment and the peak of emotional stress. Nevertheless, reducing preoperative parental anxiety remains essential — not only to create a more supportive environment and improve the child's well-being but also as a potential quality marker for paediatric anaesthesia (Olbrecht et al. 2022). 

Identifying predictors of preoperative anxiety is a key focus of current research (Cheng et al. 2023, Matava et al. 2025). Analysing generalizable data to assess their influence is therefore of great interest. While previous studies have linked younger age to increased preoperative distress (Fortier et al. 2010), our findings did not replicate this association, possibly due to the specific age range examined. The relationship between socioeconomic status and anxiety remains inconsistent across studies. While some research links low socioeconomic status to higher anxiety (Moura et al. 2016; Malik et al. 2018), studies in economically stable regions found no correlation (Davidson et al. 2006; Eijlers et al. 2021). Our findings align with the latter, suggesting that healthcare access and social integration in Germany may buffer socioeconomic-related anxiety disparities. 

IV induction was associated with lower anxiety compared to inhalation induction, diverging from previous studies (Aguilera et al. 2003). This may reflect a pre-selection of calmer and though less anxious children for IV induction in our setting, where inhalation induction is often used in children who refuse IV line placement, potentially contributing to higher anxiety rating in this group.

Despite its pragmatic approach, this study has several limitations. Anxiety was assessed solely through an observational scale (mYPAS-SF) rather than self-reported measures, which might have provided deeper insights. Additionally, asking about anxiety may itself trigger distress in both parents and children. However, parents and children were given the opportunity to discuss any concerns with the study team and the clinical staff at any time. Factors such as mental overload and periprocedural pain may influence cooperativity, potentially affecting the accuracy of mYPAS-SF scoring. Beyond anxiety, other aspects — such as patient satisfaction, autonomy, and postoperative outcomes — might be more relevant for assessing overall well-being (Rodriguez et al. 2019; Rappold et al. 2024). 

Future research should integrate tools like the Induction Compliance Checklist (ICC) and objective biomarkers (e.g., heart rate variability, cortisol) to validate anxiety assessments and improve the predictive accuracy of clinical models (Kain et al. 1997; Massoth et al. 2024). Another limitation is that anaesthesiologists conducting preoperative evaluations could not fully predict the course of anaesthesia induction, as the choice of induction method and non-pharmacological strategies ultimately depended on the anaesthesiologist in the operating theatre.

In this study, the responses to the open-ended question asking children to name a comfort place to identify individualized associations of safety were recorded but not used for any specific intervention or targeted analysis. The intention was to explore associations that may later serve as the basis for targeted interventions to reduce preoperative anxiety, as suggestions of safety have been shown to effectively decrease anxiety in surgical and other stress-related contexts (Schmidt et al., 2021; Schmidt et al., 2024; Motz et al., 2025). Similarly, the question regarding the perceived magnitude of the planned procedure showed no association with the primary or secondary outcomes, but it illustrates that parents bring concerns into the preoperative consultation. Addressing these worries may represent a potential target for indirectly reducing children’s anxiety in future studies as well.

The generalisability of our findings may be limited by the single-centre design and the specific age range of the patient population in a university hospital setting. However, the diverse sociodemographic backgrounds of the participants and the pragmatic, real-world approach support external validity. Still, caution is warranted when applying the results to settings with different clinical routines, cultural contexts, or patient characteristics.

Predicting preoperative anxiety in children remains challenging, with parents outperforming anaesthesiologists but still lacking precision. Children provided valuable insights for prediction through a simplified approach. Future efforts should prioritize involving both parents and children in anxiety management and decision-making to enhance patient-centered care. Individual factors, such as preexisting anxiety, should receive greater attention in both clinical practice and research. Future studies should focus on developing and validating multidimensional prediction models to enhance preoperative care and outcomes.

## Supplementary Information

Supplementary Material 1.

Supplementary Material 2.

## Data Availability

The datasets generated and/or analyzed during the current study are available from the corresponding author upon reasonable request.
