# Supplementary material for: Prediction of anxiety in children aged 7–12 during preoperative anaesthesia evaluation – a prospective observational study
Source: Perioper Med (Lond). 2026 Mar 12;15:42. doi: 10.1186/s13741-026-00669-2 (PMC13130822; doi:10.1186/s13741-026-00669-2)
Supplement: Supplementary file 1 — Supplementary Material 1. [file 13741_2026_669_MOESM1_ESM.docx]

Supplemental Table 1 Evaluation of Children's Feel-Good Places

| **Category** | **Subcategory** | **Total N=96** |
| --- | --- | --- |
| Home related | At home | 32 (33%) |
|  | In my bed | 8 (8%) |
|  | In child's room | 3 (3%) |
|  | On the sofa | 2 (2%) |
| Family related | With mum/dad/parents | 11 (11%) |
|  | With grandmother | 3 (3%) |
|  | With friends | 3 (3%) |
|  | With family | 1 (1%) |
| Location related | In school | 7 (7%) |
|  | On holidays | 6 (6%) |
|  | In my home country | 3 (3%) |
| Hobby/activity related | In the swimming pool | 4 (4%) |
|  | In a play center | 2 (2%) |
|  | In the riding-stable | 2 (2%) |
|  | At the soccer field | 2 (2%) |
|  | Youth center | 1 (1%) |
|  | Watching TV | 1 (1%) |
|  | In the nature | 1 (1%) |
| Other | Everywhere | 2 (2%) |
|  | No special place | 2 (2%) |

Values are presented as number (proportion). Multiple answers were possible.
