# Supplementary material for: Prediction of anxiety in children aged 7–12 during preoperative anaesthesia evaluation – a prospective observational study
Source: Perioper Med (Lond). 2026 Mar 12;15:42. doi: 10.1186/s13741-026-00669-2 (PMC13130822; doi:10.1186/s13741-026-00669-2)
Supplement: Supplementary file 2 — Supplementary Material 2. [file 13741_2026_669_MOESM2_ESM.docx]

Supplemental Table 2 Used Non-pharmacological Interventions

| **Intervention** | **Total N=140** |
| --- | --- |
| Parental Presence | 37 (26%) |
| Involvement of music | 19 (14%) |
| Clown | 17 (12%) |
| Toy | 15 (11%) |
| Video | 7 (5%) |
| Suggestion/distraction | 6 (4%) |
| Games | 3 (2%) |
| Hypnosis | 1 (1%) |
| Miscellaneous | 5 (4%) |

Values are presented as number (proportion). Multiple answers were possible.
